# Meprin β is specifically inhibited by its cognate synthetic propeptide

**DOI:** 10.1007/s00018-026-06361-4

**Published:** 2026-07-23

**Authors:** Fred Armbrust, Hannah Bruhn, Kira Bickenbach, Nicolas Steen, Sapir Shcoory, Nikolaos Afratis, Nele Korn, Hagen Körschgen, Catherine Moali, Claus U. Pietrzik, Irit Sagi, Christoph Becker-Pauly

**Affiliations:** 1 Biochemical Institute, Unit for Degradomics of the Protease Web, Kiel University, Otto-Hahn-Platz 9, Kiel, 24118 Germany; 2 Dept. of Immunology and Regenerative Biology, Weizmann Institute of Science, Max and Lillian Candiotty Building, Room 207, 234 Herzl St. Rehovot, Rehovot, 76100 Israel; 3 Department of Agricultural Development, Agri-food & Management of Natural Resources, National and Kapodistrian University of Athens, Evripos Campus, Psachna, Evia 34400 Greece; 4 Institute of Pathobiochemistry, University Medical Center Mainz, Mainz, Germany; 5 Tissue Biology and Therapeutic Engineering Laboratory (LBTI-UMR 5305 CNRS), Lyon University, Lyon, France; 6 Molecular Neurodegeneration, Institute of Pathobiochemistry, University Medical Center Mainz, Mainz, Germany

**Keywords:** Protease, Metalloprotease, Inhibitor, IL-6R, Latrophilin-3, Amyloid precursor protein, Inflammation, Alzheimer’s disease

## Abstract

Meprin β is a zinc-dependent metalloprotease of the astacin family highly expressed in kidney and intestine. Dysregulation of meprin β expression and activity was observed in several pathological conditions such as Alzheimer’s disease, cancer, fibrosis, and inflammatory bowel disease. Therefore, inhibition of meprin β could be a promising therapeutic option to treat pathological conditions influenced by this protease. However, available small-molecule compounds such as the broad-spectrum metalloproteinases inhibitors marimastat or actinonin lack specificity. Hence, more selective meprin β inhibitors needs to be developed. Here, we provide evidence that the synthetically generated cognate meprin β propeptide serves as highly specific inhibitor for the protease (IC_50_ = 1.35 µM), while other related metalloproteases such as meprin α and a disintegrin and metalloproteinases (ADAMs) are not affected. Employing a targeted approach revealed that removal of the last four C-terminal amino acids of the propeptide significantly improved its inhibitory capacity (IC_50_ = 0.68 µM). In cell-based experiments, treatment with the propeptide resulted in diminished meprin β activity and decreased cleavage of its substrates, including the interleukin-6 receptor (IL-6R) and the G protein-coupled receptor latrophilin-3 (LPHN3). Suitability was further confirmed in ex vivo cultivated organotypic brain slices, where the administration of the propeptide also resulted in decreased meprin β activity and diminished latrophilin-3 cleavage. Thus, not only the synthetic wildtype propeptide but especially a C-terminally truncated propeptide of meprin β represent highly specific inhibitors with potential for therapeutic application.

**Supplementary Information:**

The online version contains supplementary material available at https://doi.org/10.1007/s00018-026-06361-4 .

## Background

Meprin β, a zinc-dependent metalloprotease of the astacin family, is highly expressed in the kidney and intestine [1]. While its physiological role in the kidney has not been fully addressed yet, meprin β has been shown to play a crucial role for the host-microbiome homeostasis in the intestine by cleaving mucin-2, a key component of the mucus layer, and the bacterial agglutination factor galectin-3 [2, 3]. Thus, dysregulation of meprin β in the gut is associated with bacterial overgrowth and chronic intestinal inflammation [4]. Moreover, meprin β is tissue-specifically upregulated in different pathological conditions such as fibrosis [5] or Alzheimer’s Disease (AD) [6, 7], where it cleaves procollagens and the amyloid precursor protein (APP), respectively. Increased meprin β activity additionally contributes to inflammatory processes by releasing the soluble interleukin-6 receptor (IL-6R) through ectodomain shedding and thereby inducing pro-inflammatory IL-6 trans-signaling [8]. Given its involvement in these pathological conditions, targeting meprin β could be a promising therapeutic approach. However, developing applicable and selective inhibitors has proven challenging. On the one hand, hydroxamate-based metalloproteinase inhibitors such as batimastat or actinonin can be used to inhibit meprin β activity [9]. However, their use as therapeutic meprin β inhibitors would presumably come along with detrimental side effects resulting from their lack of specificity [10, 11]. On the other hand, mammalian plasma fetuin-B was identified as potent endogenous meprin β inhibitor. However, its therapeutic potential is limited by its relatively large size of 40 kDa and the inhibition of other proteases of the astacin family [12]. A more promising approach involved the development of a transition-state analog inhibitor (named F2) featuring an acidic amino-acid moiety surrounding a non-cleavable phosphinic group, which demonstrated good affinity for inhibiting meprin β [13]. However, the synthesis of this compound is challenging and both time- and cost-intensive.

The aim of this study was to investigate whether the synthetically produced cognate propeptide of meprin β has sufficient specificity and bioavailability and is therefore suitable as a potential therapeutic drug. A similar approach has been successfully conducted with the metalloproteases ADAM10 and ADAM17. It has been shown that the addition of their recombinantly expressed prodomains lead to the inhibition of protease activity [14, 15]. Interestingly, in a tumor mouse model, the intravenous injection of the recombinant prodomain of ADAM17 markedly reduced tumor burden in the lung, and partially in liver and kidney [16].

## Methods

### Recombinant enzymes and inhibitors

Recombinant meprins were obtained as described before [17]. Bone morphogenetic protein 1 (BMP1) was produced in HEK293-EBNA cells as described in [18]. Fetuin-B was purified as described before [19]. Actinonin (A6671) was purchased from Sigma Aldrich. Propeptide variants (Table 1) were generated by Genosphere BT. For the experiment comparing the inhibition of wildtype (WT) meprin β and meprin β R238Q, both meprin variants were immunoprecipitated with flag tag antibody (20543-1-AP, proteintech) and Protein G Dynabeads (Thermo Fisher Scientific) from the lysate of transfected HEK cells. For a detailed protocol see [13].

**Table 1:** Sequences of meprin β propeptide variants

Name	Sequence
propeptide	TPENFDVDGGMDQDIFDINEGLGLDLFEGDIRLDRAQIR
propeptideΔN	QDIFDINEGLGLDLFEGDIRLDRAQIR
propeptideΔC	TPENFDVDGGMDQDIFDINEGLGLDLFEGDIRLDR
propeptideΔN/C	QDIFDINEGLGLDLFEGDIRLDR
propeptideΔC D36A	TPENFDVDGGMDQAIFDINEGLGLDLFEGDIRLDR

### Microscale thermophoresis

Microscale thermophoresis (MST) was used as a fluorescent-based method to evaluate protein-ligand interactions [20]. MST works as follows: A fluorescent-labeled protein is incubated with different concentrations of a potential ligand and fluorescence intensity, measured in arbitrary units, is detected over time while a temperature gradient is applied, impacting the migration of the fluorescent-labeled protein, which depends on ligand binding. The ratio F_hot_/F_cold_ yields the normalized fluorescence, F_norm_, which is plotted against the logarithmic concentrations of the ligand. Pro- and mature meprin β were labeled with NT-647 according to the manufacturer’s instructions. The labeled meprin β (30 nM) was incubated with different concentrations of the synthetically produced propeptides ranging from 6 pM to 50 µM. Subsequently, mixtures were transferred to capillaries and measured using the Monolith NT.115 device (NanoTemper Technologies GmbH). The dissociation constant (K_D_) was calculated using the NanoTemper software.

### Cell culture, transfection and lysis

HEK293T cells and HEK293T cells deficient for ADAM10 and ADAM17 (HEK293T_A10/17^−/−^) [21] were maintained at 37 °C in an atmosphere of 5% CO_2_ in Dulbecco´s modified Eagles´s medium (DMEM; Thermo Fisher Scientific) supplemented with 10% fetal bovine serum (FBS; Thermo Fisher Scientific). Transfection with plasmid DNA, pre-mixed with polyethyleneimine (PEI) (1:3) in serum-free medium was performed at 80–90% cell confluence. Plasmid DNA containing cDNA for human soluble ADAM10 ectodomain, mouse ADAM10, human BMP1, N-terminal HA-tagged mouse latrophilin (latrophilin-3), human meprin α, human meprin β wt, human meprin β R238Q, human IL-6R, human APP695 and pcDNA3.1 as empty vector control in different combinations were added together with transfection reagent to the cell culture medium. After 24 h, the cell medium was changed to serum-free DMEM. For cell surface activity measurements, after another 24 h cells were washed with phosphate-buffered saline (PBS) and counted using a Neubauer counting chamber. If indicated, the propeptide or its C-terminally truncated variant (propeptideΔC) was added at the specified concentrations concurrently with serum removal. After an initial incubation period of 3 h, the same concentration of the propeptide variant was added again for an additional 3 h. The cells were harvested, washed with PBS and then incubated in lysis buffer (1% (v/v) Triton X-100 in PBS (pH 7,4) with cOmplete™ protease inhibitor cocktail (Roche), 1% (v/v) Triton X-100 in PBS (pH 7,4)) for 30 min at 4 °C. Lysates were centrifuged at 15,000 x g at 4 °C, cell debris was discarded. The protein concentration in the lysates was determined using the BCA assay kit (Thermo Fisher Scientific) according to manufacturer’s instructions.

### SDS-PAGE and western blot analysis

Cell lysates or supernatants were incubated for 10 min at 95 °C with sample buffer (50 mM Tris-HCl (pH 6.8), 2% (w/v) sodium dodecyl sulphate (SDS), 0.1% bromophenol blue (Merck), 10% (v/v) glycerol, 100 mM dithiothreitol (DTT)). The protein separation was performed by SDS-PAGE (120 V, 90 min) using the Mini-PROTEAN^®^ system (Bio-Rad). Western blot was accomplished with a tank-blot system (Bio-Rad) onto PVDF membranes (0.8 A, 2 h, 4 °C). Afterwards, the membranes were blocked with 5% milk (w/v) in TBS for 1 h at room temperature. The primary antibodies against glyceraldehyde 3-phosphate dehydrogenase (GAPDH) (14C10, Cell Signaling, dilution 1: 5,000), HA-tag (C29F4, Cell Signaling), meprin β (polyclonal antibody, generated against a peptide from the meprin/A5 protein/receptor protein tyrosine phosphatase µ (MAM) domain (Pineda)), latrophilin-3 (B-6, Santa Cruz), IL-6R (4–11, kindly provided by Stefan Rose-John (Kiel, Germany)), APP/sAPPα (6E10, Thermo Fisher), sAPPβ + Asp (Pineda, [22]) unless not stated differently in the respective figure legend, were incubated using a dilution of 1:1,000 with the membrane over night at 4 °C. Horseradish peroxidase-conjugated secondary antibodies (Jackson ImmunoResearch) were diluted in TBS-T (TBS with 0.1% (v/v) Tween^®^ 20) and incubated with the membranes for 1 h at room temperature. The chemiluminescence signal was detected in the Intelligent Dark Box (Fujifilm) or Amersham ImageQuant™ 800 (Cytiva) using the Super Signal^®^ West Pico/Femto Kits (Thermo Fisher Scientific) according to manufacturer’s instructions. Western blot signals were quantified with ImageJ.

### Fluorogenic peptide-based activity assay

The activity of recombinant proteases, proteases on the cell surface and proteases in conditioned supernatants were measured with well-established quenched fluorogenic peptide substrates summarized in Table 2.

**Table 2:** Peptide substrates. The final concentration in the assays is indicated in the c_substrate_ column. The references column summarizes successful use of the particular fluorogenic peptides to show peptidase activity of the respective protease. ADAM10 substrate was obtained from BioZyme Inc., MMP1/2/14 substrate from R&D Systems and ACE1/ACE2/ADAM17 substrates from Sigma Aldrich, ovastacin substrate from Biosyntan and the others from Genosphere BT. 5-fam: 5-carboxyfluorescein, dabcyl: Acetyl-dArg(3)-dGlu(3)-hexaminoyl-K-4-((4-(Dimethylamino)phenyl)azo)benzoic, mca: 7-methyloxycoumarin-4-yl, dnp: K-ε-dinitrophenyl

Substrate for	Sequence	^λ^exitation	^λ^emission	^c^substrate	References
ADAM10	(dabcyl)-PRYEYKMGK-(5-fam)	480 nm	530 nm	10 µM	[23]
BMP1	(mca)-YVADAPK-(dnp)	320 nm	405 nm	20 µM	[24]
Meprin α	(mca)-HVANDPIW-(dnp)	320 nm	405 nm	20 µM	[3]
Meprin β	(mca)-EDEDED-(dnp)	320 nm	405 nm	25 µM	[1]
Meprin β R238Q	(mca)-EDAEDA-(dnp)	320 nm	405 nm	25 µM	[13]
MMP1, MMP2, MMP14	(mca)-PLGL-(dpa)-AR-NH_2_	320 nm	405 nm	20 µM	[25]
ACE1	(mca)-RPPGFSAFK-(dnp)	320 nm	405 nm	10 µM	[26]
ACE2	(mca)-APK(Dnp)-OH	320 nm	405 nm	10 µM	[27]
ADAM17	(abz)-LAQAVRSSSR-(dpa)	320 nm	405 nm	10 µM	[28]
Ovastacin	Ac-RE(Edans)‐DR‐Nle‐VGDDPYK(Dabcyl)‐NH_2_	350 nm	520 nm	25 µM	[29]

For this purpose, 5 nM of recombinant enzymes, 500,000 cells or up to 90 µl conditioned supernatant was transferred in 96-well plates in a total volume of 100 µl PBS or DMEM only. For the measurement of meprin α and meprin β in conditioned medium, the enzymes were proteolytically activated with 5 µg/ml trypsin (Sigma Aldrich) and trypsin was afterwards inhibited by ovomucoid (Sigma Aldrich) both steps for 10 min at 37 °C. For activity measurement of human angiotensin converting enzyme (ACE1, Sigma-Aldrich) 50 mM HEPES, 300 mM NaCl (pH 8.0), for human ACE 2 (Sigma-Aldrich) 50 mM Tris, 150 mM NaCl (pH 7.5) and for MMP1/2/14 (R&D) 50 mM HEPES, 10 mM CaCl_2_ (pH 7.5) were used as buffer. Murine ovastacin was heterologously expressed and purified as previously described [30]. Activation was achieved with human plasmin (Haematologic Technologies). Enzyme kinetic measurements were performed as described [29]. If indicated in the figure, additional inhibitors, meprin β propeptide variants, fetuin-B, or actinonin, were added and preincubated for 10 min at 37 °C. Afterwards, the respective peptide substrate was added as depicted in Table 2. The measurement was conducted at 37 °C for 1–2 h. Every 30 to 60 s, the fluorescence at the wavelengths indicated in Table 2 was monitored. Relative fluorescent units (RFUs) were normalized to the respective control samples only containing the respective background medium and peptide substrate. Negative values were set to 0. The bar graphs display the slope of the linear gain of fluorescence within the linear range (max. first 30 min). K_i_ (inhibition constant) values were determined using GraphPad Prism.

### Preincubation and stability testing of propeptide inhibitor

To analyse stability properties of propeptideΔC pre-incubations at a concentration of 20 µM at different temperatures for 20 min, freeze-thrawn for indicated times or preincubated using PBS with different pH values for 20 min (buffer only as a control, measurement of meprin β activity after adjustment to pH 7.4). To test proteolytic stability propeptideΔC (20 µM) was incubated with trypsin (1–5 µg/ml) in PBS, chymotrypsin (EDQM) (1–5 µg/ml) in 100 mM Tris, 10 mM CaCl_2_ (pH 7.8) or thrombin (Sigma) (1–5 µg/ml) in PBS for 20 min at 37 °C or in case of chymotrypsin at 30 °C. To inhibit trypsin/chymotrypsin afterwards PMSF (phenylmethylsulfonyl fluoride) (final 1 mM) was added. To test stability in mouse serum, mouse serum from C57BL/6 mice were incubated in a 1:1 ratio at 37 °C with 200 µM propeptideΔC for 20 min. Corresponding incubation conditions without propeptideΔC were used as controls for subsequent activity assays.

### Experimental animals and Generation, cultivation and lysis of organotypic brain slice cultures (OBSCs)

Mice were maintained under constant environmental conditions (12-hour light-dark cycles, temperature) and in an individually ventilated cage system with food and drinking water ad libitum in accordance with the ethical standards and current guidelines of the European Union. The animals were kept solely for organ removal. This is not an animal experiment in the sense of the German Animal Welfare Act, as no animal was stressed or manipulated. Therefore, no further approval from the responsible authority (Ministry of Agriculture, Rural Areas, Europe and Consumer Protection including the Ethics Committee according to Sect.  15 of the Animal Welfare Act) was required. Meprin β knock-in mice overexpressing meprin β with C-terminal HA tag in astrocytes were generated [22]. Rosa26^*Mep1b−HA*^ mice, which were heterozygous for GFAP-dependent Cre, are termed as GFAP^Cre+/−^;Rosa26^*Mep1b*−HA^ mice (or briefly Cre^+/−^). The respective Cre-negative control animals are referred to as GFAP^Cre−/−^;Rosa26^*Mep1b*−HA^ (or briefly Cre^−/−^). Mice were sacrificed by cervical dislocation and brains were isolated. OBSC generation, cultivation and lysis was conducted as described in [22]. Half of the brain slices of each genotype were randomly selected and treated with 5 µM propeptide∆C together with the serum-free medium 24 h prior harvesting, while the rest were left untreated. Additional 5 µM propeptide were added every 6 h.

### Statistical analysis and illustrations

All statistical analyses were performed with the GraphPad Prism 10 software. Conducted tests are stated in the respective figure descriptions (*: *p* < 0.05; **: *p* < 0.01; ***: *p* < 0.001). Protein structures were generated using Chimera 1.17.3. The figures were created with Microsoft PowerPoint and BioRender.com.

## Results

### Meprin β is inhibited by exogenous addition of its propeptide

**Fig. 1:**
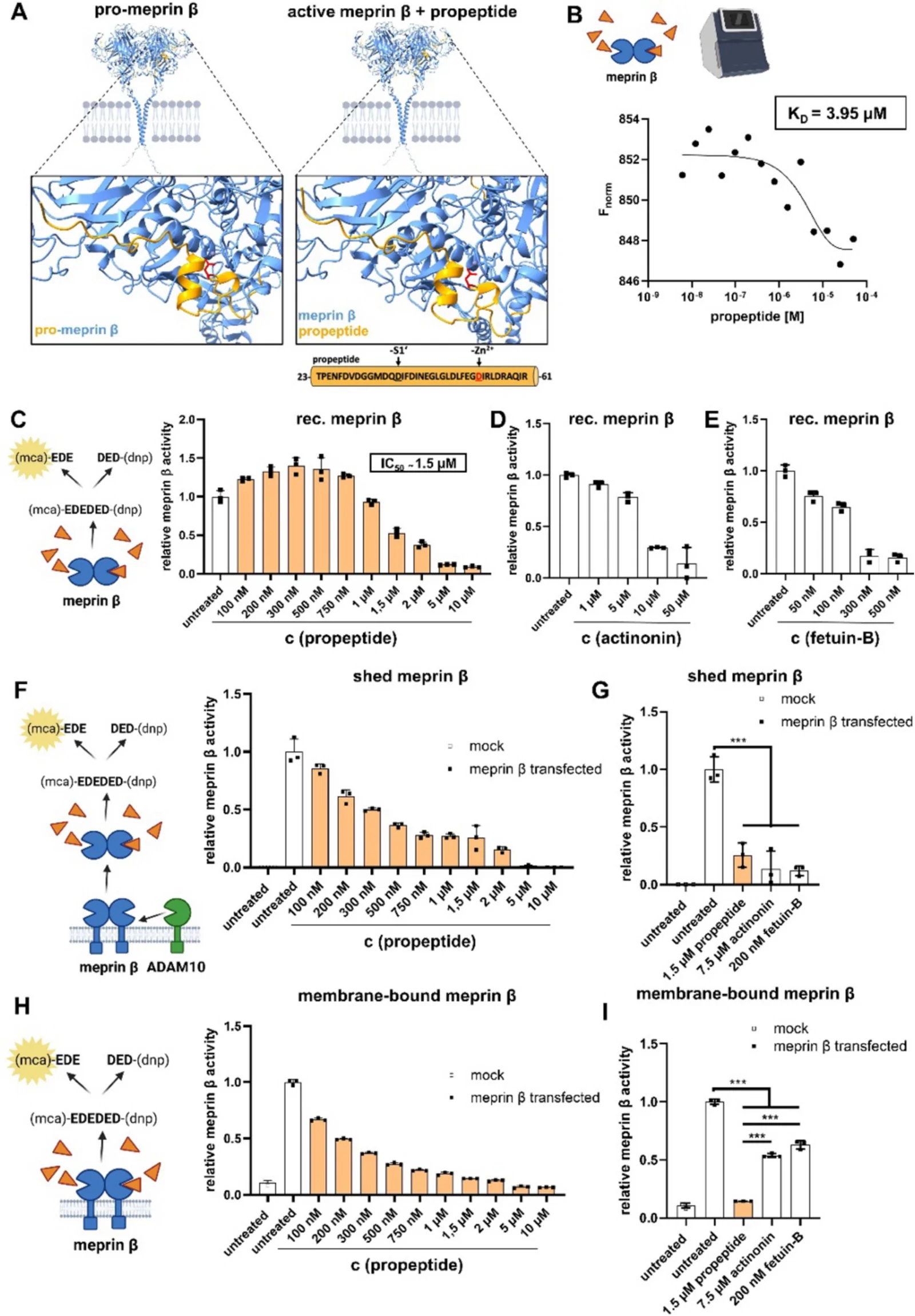
Meprin β is inhibited by its synthetic propeptide. (**A**) Protein structure models of pro-meprin β (left) compared to structure of the complex of propeptide (orange) added to active meprin β (generated with AlphaFold Server using the UniProt Consortium 2021 sequence and illustrated with ChimeraX1.9). In the sequence below, amino acids important for the interaction of the propeptide with Zn^2+^ and S1’ position within the active site are highlighted. (**B**) Recombinant meprin β (30 nM) was fluorescence labeled and incubated with different concentrations of synthetically produced propeptide ranging from 6 pM to 50 µM. The dissociation constant (K_D_) was calculated using the NanoTemper software. (**C**),(**D**),(**E**) Recombinant meprin β was preincubated with different concentrations of its synthetic propeptide, actinonin or fetuin-B. Meprin β activity was measured using a quenched fluorogenic peptide substrate (*n* = 3). IC_50_ values were calculated. The propeptide is depicted in orange. (**F**), (**G**) HEK ADAM10/17^−/−^ cells were transfected with meprin β and ADAM10. The conditioned supernatant was preincubated with different concentrations of its synthetic propeptide, actinonin or fetuin-B. Meprin β activity was measured using a quenched fluorogenic peptide substrate (*n* = 3). The propeptide is depicted in orange. The significance in (**G**) was determined by one-way ANOVA with Bonferroni adjustment (ns: *p* ≥ 0.05; ***: *p* < 0.001). (**H**),(**I**) HEK cells transfected with meprin β were preincubated with different concentrations of its synthetic propeptide, actinonin or fetuin-B. Meprin β activity at the cell surface was measured using a quenched fluorescent peptide substrate (*n* = 3). The propeptide is depicted in orange. The significance in (**I**) was determined by one-way ANOVA with Bonferroni adjustment (***: *p* < 0.001)

To analyze whether the propeptide of meprin β serves as a specific inhibitor, we synthesized the propeptide based on the amino acid sequence annotated in uniprot (Fig. 1A). From AlphaFold modeling, the complex of active meprin β together with the synthetic cognate propeptide clearly resembles the structure of pro-meprin β, where the propeptide is covalently linked to the enzyme with only little differences in the very C-terminal part of the propeptide. MST measurements were conducted to analyze the interaction between recombinant meprin β and the synthetically produced propeptide and the sigmoidal curve obtained validated an interaction with a K_D_ of 3.95 µM (Fig. 1B and Supplementary Figure S1A). As negative control, the propeptide was additionally applied to pro-meprin β, which showed no interaction as indicated by the absence of a sigmoidal curve (Supplementary Figure S1B).

To evaluate the inhibitory potential of the propeptide, we added various concentrations to recombinant meprin β in a fluorescent-based peptide cleavage assay (Fig. 1C). In the micromolar range, we observed a concentration-dependent decrease in activity, up to 90% inhibition with 10 µM propeptide. We also analyzed the known meprin β inhibitors actinonin and fetuin-B in the same experimental setup and estimated IC_50_ values of about 7.5 µM for actinonin and 200 nM for fetuin-B (Fig. 1D and E), which is in the range of previous observations [9, 12, 31]. To investigate the inhibitory capacity of the propeptide towards proteolytically shed meprin β, we analyzed soluble meprin β from conditioned HEK cell supernatants (Fig. 1F). Therefore, we transfected HEK ADAM10/17^−/−^ cells with meprin β and ADAM10. Of note, ADAM10 and ADAM17 are sheddases of meprin β releasing the ectodomain of meprin β to the supernatant, which was also the case upon retransfection of ADAM10. Of note, in the conditioned medium meprin β activity was completely abolished by addition of the propeptide in a concentration-dependent manner. To assess inhibition of meprin β upon application of the propeptide, actinonin and fetuin-B to the conditioned medium, we determined the necessary concentrations for each individual inhibitor leading to a similar reduction of meprin β activity of approximately 75% (Fig. 1G).

Regarding pathological conditions, the inhibition of increased meprin β activity, especially in the membrane-bound state, is important. Therefore, we quantified meprin β cell surface activity using transfected HEK cells (Fig. 1H). We observed that membrane-bound meprin β was concentration-dependently inhibited by its propeptide, down to 10% remaining activity at a concentration of 10 µM propeptide. To further investigate the inhibition of membrane bound meprin β by the propeptide, actinonin, and fetuin-B, we applied these inhibitors at concentrations previously established in Fig. 1G (Fig. 1I). Notably, the propeptide resulted in meprin β activity inhibition of 80%, while actinonin and fetuin-B revealed only inhibition of about 40%. In terms of inhibiting the membrane-bound form of meprin β, these results indicate that the inhibitory potency of the propeptide exceeds that of actinonin and fetuin-B. Additionally, we examined the effects of meprin β inhibition using HEK ADAM10/17^−/−^ cells and observed similar results (Supplementary Figure S2A, B).

### The inhibition of meprin β by its synthetic propeptide is highly specific

**Fig. 2:**
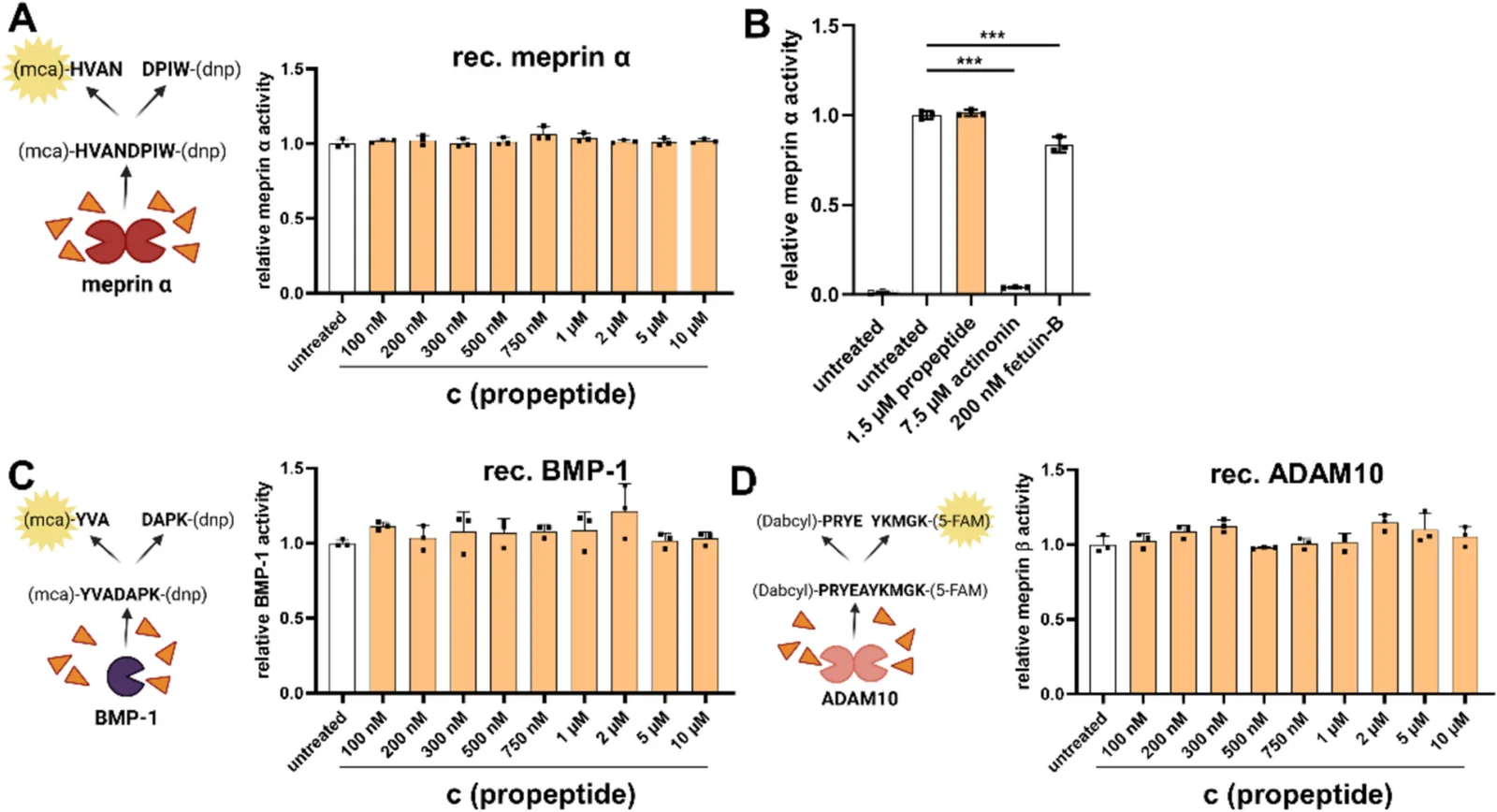
The inhibitory capacity of the synthetic propeptide is specific for meprin β. (**A**), (**B**) Recombinant meprin α (5 nM), (**C**) recombinant BMP1 (5 nM) or (**D**) recombinant ADAM10 (5 nM) was preincubated with different concentrations of the synthetic propeptide of meprin β, actinonin or fetuin-B. The meprin α or BMP1 activity was measured using a quenched fluorescent peptide substrate (*n* = 3). The propeptide is depicted in orange. The significance in (**B**) was determined by one-way ANOVA with Bonferroni adjustment (ns: *p* ≥ 0.05; ***: *p* < 0.001)

To assess the specificity of the meprin β propeptide as an inhibitor, we additionally tested other related proteases. Therefore, we examined meprin β’s closest relative meprin α, BMP1 - another member of the astacin protease family group, and ADAM10, which share a similar substrate pool with meprin β. Peptide cleavage assays revealed that the propeptide of meprin β is not capable of inhibiting recombinant meprin α (Fig. 2A). This finding was further confirmed with HEK cell-derived meprin α, which, however, could be inhibited by actinonin and fetuin-B (Fig. 2B). Additionally, recombinant BMP1 (Fig. 2C) and ADAM10 (Fig. 2D) were not inhibited by the propeptide, demonstrating the remarkable specificity of the meprin β propeptide as an inhibitor.

### The inhibitory capacity of the propeptide is increased by truncation of the C-terminus

**Fig. 3:**
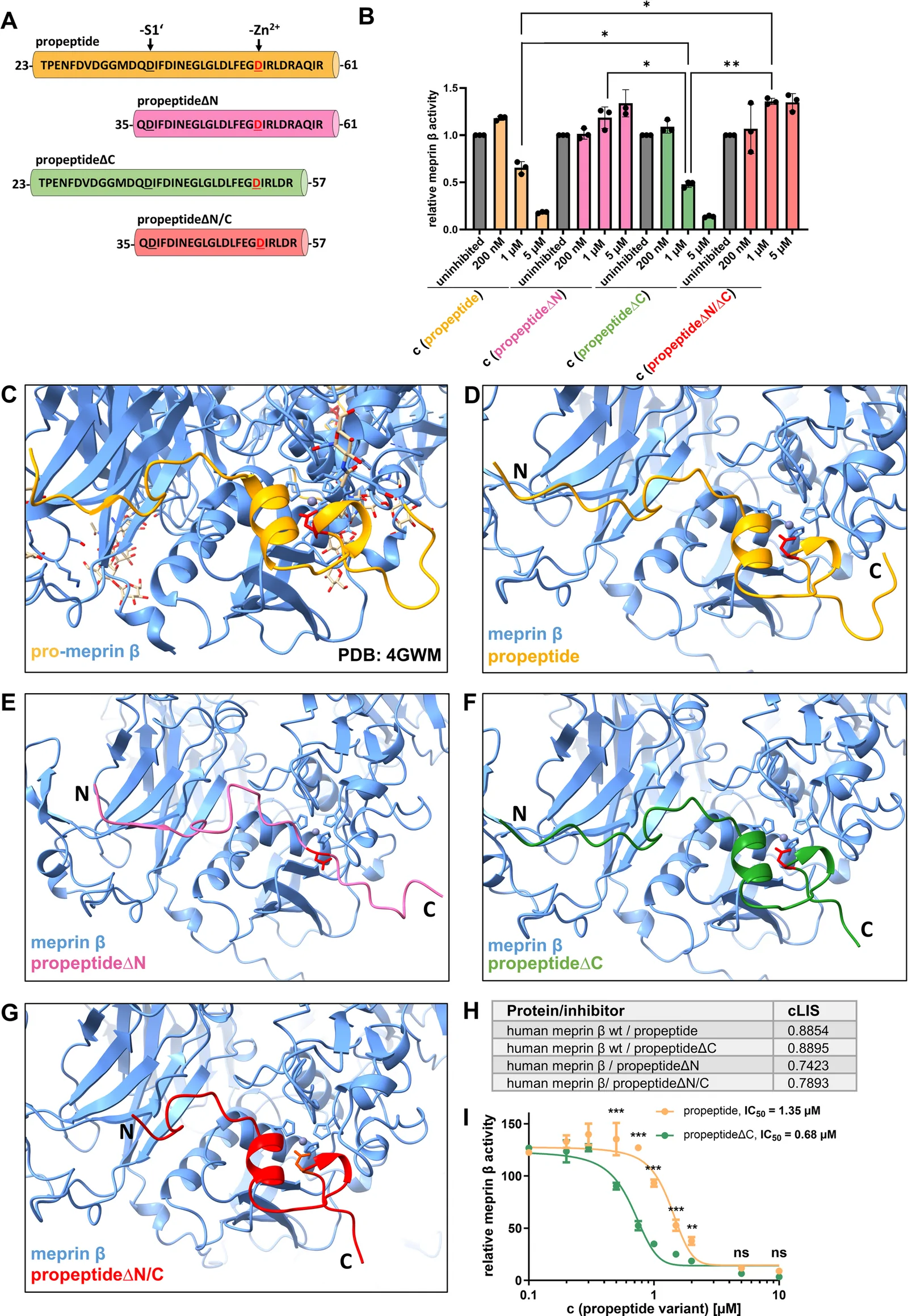
The inhibitory capacity of the propeptide is increased by truncation of the C-terminus. (A) Amino acid sequences of propeptide variants are depicted. D36, located on the bottom of the S1’ site, and D52 (red), binding the zinc ion in the active site, are underlined. (B) Recombinant meprin β (5 nM) was preincubated with different concentrations of propeptide variants as depicted in (A). The meprin β activity was measured using a quenched fluorogenic peptide substrate (n = 3). The significance was determined by two-way ANOVA with Bonferroni adjustment (ns: p ≥ 0.05; ***: p < 0.001). (C) Crystal structure of dimeric pro-meprin β (PDB: 4GWM)32 with close-up view on the propeptide. (D-G) Protein structure models of meprin β together with indicated propeptide variants from (A). Protein structures were generated with AlphaFold Server using the UniProt Consortium 2021 sequence (UniProt ID: Q16820) and visualized using ChimeraX1.9. (I) Local interaction score in contact interface (cLIS) of the interaction of meprin β together with different propeptide variants, calculated as described in 33. (H) Recombinant meprin β was preincubated with different concentrations of propeptide and propeptide∆C. Meprin β activity was measured using a quenched fluorogenic peptide substrate (n = 3). The significance was determined by two-way ANOVA with Bonferroni adjustment (ns: p ≥ 0.05; **: p < 0.001; ***: p < 0.001)

In a targeted approach, we aimed to investigate how truncation of the termini influences the inhibitory capacity of the propeptide. Given the significance of D36 in its interaction with R238 and the crucial role of D49 in binding to the zinc ion within the active site cleft [13], we preserved these residues and their neighboring amino acids (Fig. 3A). Notably, we observed that truncating the C-terminus resulted in significant increase of the inhibitory capacity (Fig. 3B). Surprisingly, when the N-terminus was removed, no inhibitory capacity of this truncated propeptide could be observed anymore. As previously demonstrated in an experimentally solved crystal structure of pro-meprin β (PDB: 4GWM) [32] (Fig. 3C), the N-terminal part of the propeptide is in close proximity to both the catalytic and tumor necrosis factor receptor-associated factor (TRAF) domain of meprin β, harboring critical interaction sites for propeptide binding (Fig. 3C-G). In contrast, the C-terminal loop of the propeptide protrudes away from the catalytic domain of meprin β. As a result, no crucial interaction sites are present in this region, and its deletion seems to confer a sterical advantage. Calculation of local interaction score in the contact interface (cLIS, normalized between 0 (very low confidence of interaction) and 1 (very high confidence of interaction) as described in [33]) further supports these findings, as cLIS was decreased for propeptide ∆N and propeptide ∆N/C compared to propeptide und propeptide∆C (Fig. 3H). Consequently, this led to a decrease in the IC_50_ from 1.35 µM for the wild-type propeptide to 0.68 µM for propeptide∆C (Fig. 3I).

To assess the specificity of propeptideΔC, we analyzed its potential inhibitory effects on a panel of other metalloproteases, including angiotensin-converting enzyme 1 (ACE1), ACE2, ADAM17, MMP1, MMP2, MMP14, and ovastacin (Supplementary Figure S3). No effect of propeptideΔC on the proteolytic activity of these metalloproteases was observed, with the exception of ovastacin, whose activity was significantly reduced at propeptideΔC concentrations of ≥ 1 µM.

### The synthetic propeptide is a weak inhibitor of the cancer associated meprin β R238Q variant

**Fig. 4:**
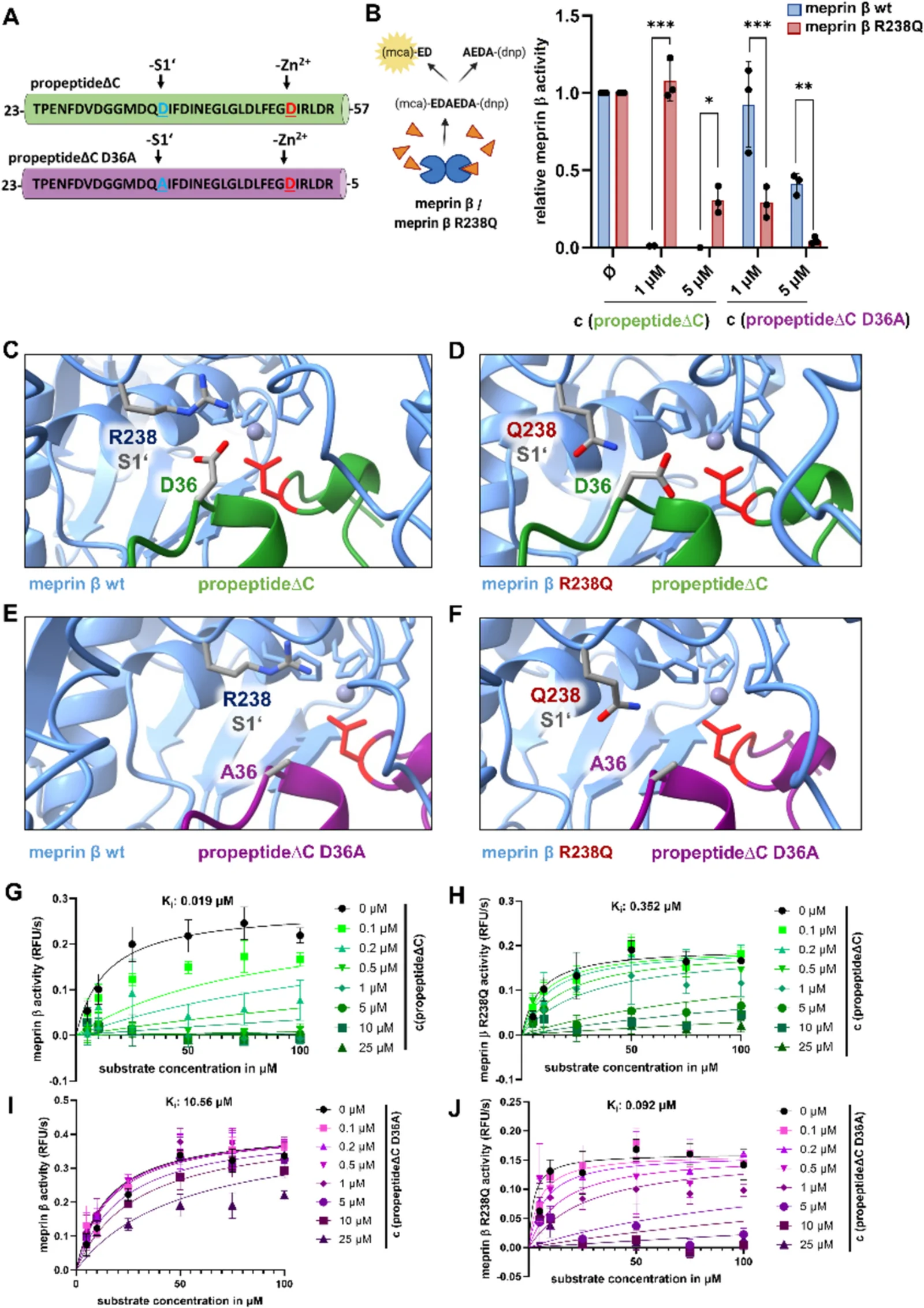
Optimization of the propeptide for the inhibition of the cancer-associated variant meprin β R238Q. (**A**) Sequences of propeptide variants (propeptideΔC and propeptideΔC D36A) are depicted. For propeptideΔC D36A, the aspartate located on the bottom of the S1’ site was replaced by an alanine residue for a better inhibition of the meprin β R238Q variant. (**B**) Recombinant wt meprin β (5 nM) and the R238Q variant were preincubated with the synthetically produced propeptideΔC and propeptideΔC D36A. The meprin β activity was measured using a quenched fluorogenic peptide substrate (*n* = 3). The significance was determined by one-way ANOVA with Bonferroni adjustment (ns: *p* ≥ 0.05; ***: *p* < 0.001). The propeptide is depicted in orange. (**C-F**) Protein structure models of meprin β wt and meprin β R238Q together with propeptideΔC and propeptideΔC D36A. Structures were generated with AlphaFold Server using the UniProt Consortium 2021 sequence and visualised using ChimeraX1.9. (**G-J**) Recombinant wt meprin β (5 nM) and the R238Q variant were preincubated with the synthetically produced propeptideΔC and propeptideΔC D36A in different concentrations. The meprin β activity was measured using different concentrations of a quenched fluorogenic peptide substrate ((mca)-EDAEDA-(dnp)) (*n* = 3). To determine competitive inhibition enzyme activity across varying substrate and inhibitor concentrations were measured and K_i_ (inhibition constant) values were determined using GraphPad Prism [34]

We tested the inhibitory capacity of the propeptide towards the cancer-associated meprin β R238Q variant described in [13]. This variant was found in various cancer tissues and harbors a mutation in the S1’ site of meprin β. R238, located at the bottom of this specificity pocket, predominantly governs the preference for acidic amino acids and interacts with D36 of the propeptide (Fig. 4A). With the substitution R238Q in meprin β, D36 of the propeptide lacks a partner for forming an ionic interaction, potentially leading to reduced inhibition upon exogenous addition of the propeptide. Indeed, in a peptide cleavage assay we observed that application of 1 µM of the propeptide resulted in inhibition of meprin β wt by 99%, while the variant meprin β R238Q is not inhibited at all (Fig. 4B). Only when the propeptide concentration was increased to 5 µM we observed inhibition of the meprin β R238Q variant of 70%. This outcome underscores how specificity is enabled by intermolecular interactions between meprin β and its propeptide, in particular involving the S1’ position (Fig. 4C). To generate a propeptide inhibitor with improved efficacy against the cancer-associated meprin β R238Q variant, the aspartate at position 36 (D36) in the propeptide was replaced by a smaller alanine residue. Indeed, this leads to a more efficient inhibition of meprin β R238Q, while at the same time the inhibition of meprin β wt by propeptid∆C D36A was strongly reduced compared to propeptide∆C. This is reflected in the calculated K_i_ values obtained for the various combinations of meprin β and propeptide variants (Fig. 4G–J). Meprin β wt is efficiently inhibited by propeptideΔC, with a K_i_ of 0.019 µM, whereas the K_i_ value for inhibition of meprin β R238Q by propeptideΔC is markedly increased to 0.352 µM. The opposite effect is observed for the optimized propeptideΔC D36A variant: the K_i_ for propeptideΔC D36A for the meprin β R238Q with 0.092 µM is lower compared to 10.56 µM for meprin β wt, indicating substantially increased inhibitory potency against the cancer-associated variant.

### Substrate cleavage by meprin β is inhibited by the synthetic propeptide *in cellulo*

**Fig. 5:**
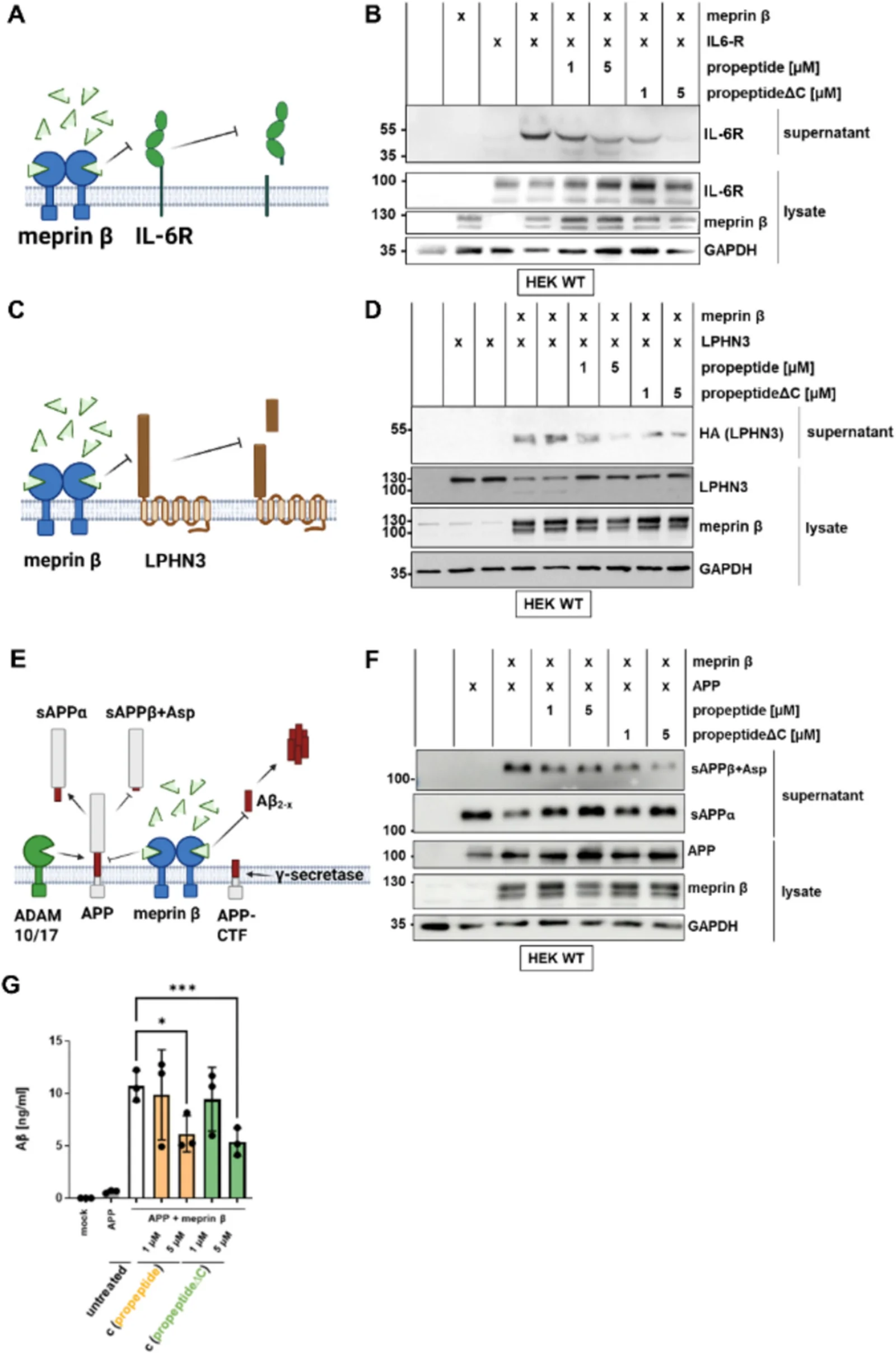
Substrate cleavage by meprin β is inhibited by the synthetic propeptide in transfected HEK cells. (**A**) Scheme showing the propeptide inhibition of meprin β-mediated IL-6R cleavage. (**B**) HEK cells were transfected with IL-6R and/or meprin β. Afterwards, the cells were treated with the indicated concentration of propeptide or propeptide∆C. The cells were lysed and proteins were analyzed in cell lysates and supernatants using SDS-PAGE and Western blot (*n* = 3). (**C**) Scheme showing the propeptide inhibition of meprin β-mediated latrophilin-3 cleavage. (**D**) HEK cells were transfected with latrophilin-3 and/or meprin β and analyzed as described in (B). (**E**) Scheme showing APP processing and propeptide inhibition of meprin β-mediated APP cleavage. (**F**) HEK cells were transfected with APP and/or meprin β and analyzed as described in (B). (**G**) Aβ_x−40_-ELISA of supernatants from cells treated as described in (F). The significance was determined by two-way ANOVA with Bonferroni adjustment (ns: *p* ≥ 0.05; **: *p* < 0.001; ***: *p* < 0.001)

To assess whether the propeptide variants also inhibit the cleavage of meprin β substrates in a cellular context, we transfected HEK cells with meprin β and the well-established meprin β substrates IL-6R [8], latrophilin-3 [35] as well as the amyloid precursor protein (APP)^6^. Transfecting HEK cells with meprin β and IL-6R, resulted in a 55 kDa cleavage fragment that accumulates in the supernatant (Fig. 5A, B). In the presence of the propeptide, the levels of the released IL-6R cleavage fragment are reduced in a concentration-dependent manner. This effect is even more pronounced when propeptide∆C was added.

Upon transfection of HEK cells with meprin β and latrophilin-3, the full-length form of latrophilin-3 in the lysate is decreased, and simultaneously, a 90 kDa cleavage fragment in the lysate and a 55 kDa fragment in the supernatant accumulates (Fig. 5C, D). In the presence of the propeptide, these proteolytic effects on latrophilin-3 were diminished in a concentration-dependent manner.

Meprin β acts as an alternative β-secretase, generating pre-dominantly N-terminally truncated Aβ_2−x_ peptides (Fig. 5E) 6, which are highly abundant in Alzheimer’s disease (AD) patients’ brains [36]. Co-transfection of meprin β and APP, compared to transfection with APP only, leads to higher amounts of sAPPβ (~ 100 kDa, detected with a neo-epitope specific antibody [22]) and a contrary effect on sAPPα fragments (~ 100 kDa), produced by α-secretases such as a ADAM10 or ADAM17 [37] (Fig. 5F). In the presence of the propeptide, and even more efficiently by the propeptide∆C, the generation of sAPPβ by meprin β was reduced in a concentration-dependent manner. Due to the competition of α-secretases and β-secretases for the processing of APP, successful inhibition of meprin β by the propeptide is also detectable by elevated sAPPα generation (Fig. 5F). Furthermore, an Aβ_x−40_-ELISA showed a significant inhibitory effect of the propeptide inhibitors on generation of neurotoxic Aβ peptides (Fig. 5G).

### Latrophilin-3 cleavage by meprin β is inhibited by the improved propeptide∆C in ex vivo cultivated brain slices

**Fig. 6:**
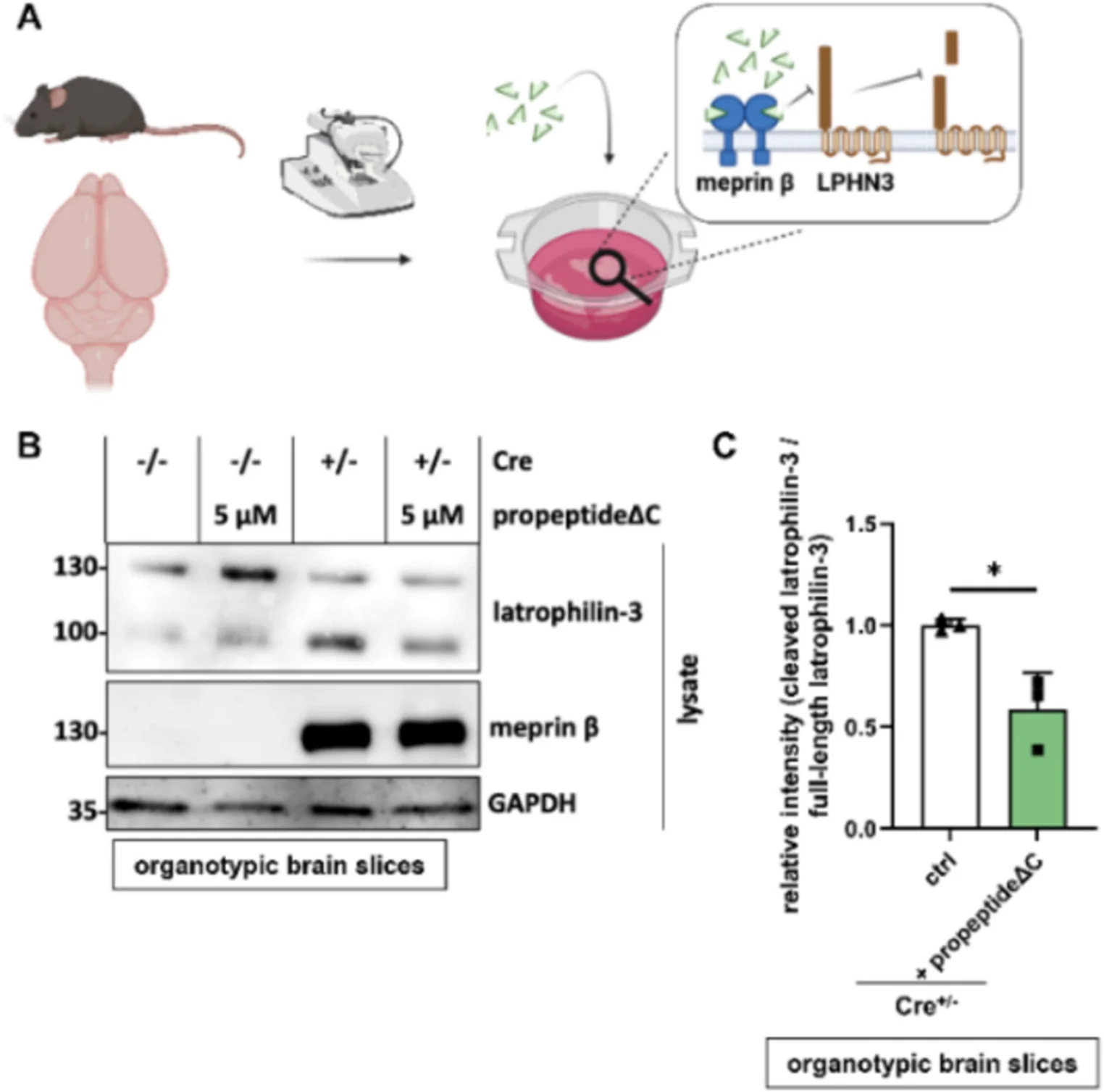
Substrate cleavage by meprin β is inhibited by the improved propeptide∆C in *ex viv*o cultivated brain slices. (**A**) Organotypic brain slices were obtained by sagittal cutting the brain into 250 μm thick slices. Slices were cultivated for 20 days and treated with propeptide∆C (**B**) Organotypic brain slices of GFAP^Cre+/−^;Rosa26^*Mep1b*−HA^ and GFAP^Cre--/−^;Rosa26^*Mep1b*−HA^ mice. Half of the brain slices of each genotype were randomly selected and treated with 5 µM propeptide∆C, whereas the rest was left untreated. The organotypic brain slices were lysed and proteins were analyzed with SDS-PAGE and Western blot (*n* = 3). (**C**) The Western blot signals for latrophilin-3 in panel (B) were quantified (*n* = 3). The intensity of the signal corresponding to the 90 kDa cleavage fragment was measured relative to that of the full-length form at 130 kDa. The significance was determined by an unpaired t-test (*: *p* < 0.05)

To determine whether inhibition of meprin β by propeptide∆C is effective in a more physiological context, we employed ex vivo cultivated brain slices. Given that the previously analyzed meprin β substrate latrophilin-3 is highly expressed in astrocytes, we utilized conditional knock-in mice that overexpress meprin β in astrocytes under the GFAP-promotor [23]. After generating the organotypic brain slices, they were cultivated and treated with propeptide∆C (Fig. 6A).

In brain lysates of the GFAP^Cre+/−^;Rosa26^*Mep1b*−HA^ mice overexpressing meprin β, the band corresponding to full-length latrophilin-3 at 130 kDa was decreased, whereas a cleavage fragment of 100 kDa was increased compared to control brain lysates. Treatment with propeptide∆C resulted in a reduction of the cleavage fragment (Fig. 6B). By quantifying the ratio between cleaved and full-length latrophilin-3, we observed a significant inhibition of meprin β-mediated latrophilin-3 cleavage by propeptide∆C (Fig. 6C). To evaluate the potential in vivo applicability of propeptideΔC as a therapeutic inhibitor, we assessed its stability under various pH and temperature conditions as well as its susceptibility to proteolytic degradation (Supplementary Figure S4). PropeptideΔC remained stable over a broad pH range (pH 1–10) and at temperatures up to 95 °C, and it also tolerated multiple freeze–thaw cycles without loss of inhibitory capacity. In contrast, pre-incubation with trypsin, and to a lesser extent with chymotrypsin or thrombin, reduced the inhibitory capacity of propeptideΔC, likely due to proteolytic processing of the inhibitor. Importantly, pre-incubation with murine serum did not affect the inhibitory capacity of propeptideΔC. Taken together, these results identified propeptideΔC as a highly stable and selective inhibitor with promising characteristics for future in vivo studies.

## Discussion

Meprin β is an astacin metalloprotease that is upregulated in several pathological conditions [1]. For instance, elevated levels of meprin β have been detected in the brains of Alzheimer’s disease patients, where it may contribute to the generation of neurotoxic Aβ peptides [6, 7]. In peripheral tissues such as skin and lung, meprin β participates in extracellular matrix remodeling and has been implicated in fibrotic processes, partly through cleavage of collagen and other matrix components [5, 8, 38]. In addition, meprin β modulates inflammatory responses through proteolytic processing of cytokines and cytokine receptors. It has been shown to activate pro-IL-1β and pro-IL-18 into their bioactive forms, thereby promoting inflammation [39]. Furthermore, meprin β can cleave the membrane-bound IL-6R, generating soluble IL-6R and enabling IL-6 trans-signaling [8]. Notably, meprins can also directly cleave and inactivate IL-6 itself, indicating a complex and context-dependent role in regulating IL-6–mediated signaling [8].To achieve a treatment with minimal side effects for pathologies associated with meprin β, the use of therapeutic inhibitors is a promising option. However, actinonin [9] and fetuin-B [12], the currently available inhibitors for meprin β, do not meet the requirements in terms of specificity and, for fetuin-B, in terms of size and accessibility the of membrane-bound protease. In this regard, peptide-based inhibitors could represent a more promising approach. A published phosphinic peptide-based F2 inhibitor for meprin β [13] has been developed, but synthesis of this compound remains challenging and time-/cost-intensive. Furthermore, such phosphinic-based peptides are more likely to cause an immune response when applied in vivo. Thus, the use of endogenously occurring peptides may represent a preferable approach.

Therefore, in this study, we characterized the synthetically produced cognate meprin β propeptide as a competitive inhibitor for the enzyme. We demonstrated that the propeptide effectively inhibits both membrane-bound and soluble forms of meprin β with an inhibition constant in the micromolar range. Other meprin β-related proteases, such as meprin α and BMP-1 from the astacin family [40], as well as ADAM10, ADAM17, MMP1, MMP2 and MMP14 from the metzincin superfamily, are not inhibited by the propeptide, demonstrating the specificity of the compound for meprin β. Importantly, also activity of ACE1 and ACE2, which colocalize with meprin β in the kidney [41], is not affected by the cognate meprin β propeptide. However, we observed that the activity of the closely-related astacin protease ovastacin, is reduced by the addition of 1 µM or higher concentrations of the meprin β propeptide inhibitor. Ovastacin, like meprins exhibits a strong preference for aspartate in P1’ position [42]. From our experiments, it remains unclear whether the observed effect results from the meprin β propeptide acting as an inhibitor of ovastacin or as a competing substrate. However, given that ovastacin expression is restricted to oocytes, the observed effect is unlikely to have a significant impact on the potential therapeutic use of the inhibitor under pathophysiological conditions [40, 42, 43]. The observed specificity is a significant improvement over commonly used meprin β inhibitors. Typically, broad-spectrum metalloproteinase inhibitors like actinonin, galardin and batimastat are employed, whose mode of function is primarily based on zinc chelation through a hydroxamate group [9, 44–46]. Furthermore, TAPI-1, and TAPI-2, also inhibit meprin β activity [9]. However, several members of the metzincin family are targeted by these inhibitors, particularly matrix metalloproteinases (MMPs), which are affected to a much greater extent than meprin β. This makes these broad-spectrum inhibitors unsuitable for therapeutic applications that specifically target meprin β. More selective hydroxamate-based inhibitors have been developed, published lately in [47–49], showing inhibition in the in the lower nanomolar range, however, still facing problems typical for hydroxamates like metabolic instability, for example in plasma, and toxic metabolic byproducts [50].

Another prominent and endogenous inhibitor of meprin β is the plasma protein fetuin-B. Fetuin-B has been shown to inhibit meprin β in the nanomolar range [12]. However, the specificity is still an issue as it also inhibits closely related proteases like meprin α or ovastacin. Another drawback of fetuin-B is the molecular weight of 40 kDa, which would lead to high production costs of the recombinant protein or batch to batch variances. Furthermore, we observed that while soluble meprin β is strongly inhibited by fetuin-B, the potency is significantly reduced towards membrane-bound meprin β. As previously discussed, inhibition by fetuin-B probably requires a molecular rearrangement of the meprin β dimer from a “membrane-distal” to a “membrane-proximal” conformation [19]. Therefore, the propeptide has advantages over fetuin-B in terms of size, specificity, and accessibility. By deleting amino acids from the N- and C-termini of the synthetic meprin β propeptide, we observed that the N-terminus, which interacts with the TRAF and catalytic domain of meprin β, is crucial for appropriate binding of the propeptide. However, removing the C-terminal loop improves the inhibitory capacity and leads to increased inhibition of substrate cleavage, as demonstrated with the well-established substrates IL-6R, latrophilin-3 and APP. IL-6R serves as the receptor for the key regulatory cytokine IL-6. Shedding of IL-6R by meprin β enables a process known as IL-6 trans-signaling, where shed IL-6R binds to IL-6 and subsequently activates signaling via gp130 on cells that do not express IL-6R [8, 51]. Excessive trans-signaling may lead to detrimental pro-inflammatory signaling [52]. Besides latrophilin-3, expressed by astrocytes, plays a crucial role in synapse formation by interacting with neuronal proteins during synapse development. Impaired latrophilin-3 function has been associated with hyperactivity in mice and attention deficit hyperactivity disorder (ADHD) in humans. Finally, upregulation of meprin β in AD patients’ brains [7] and its function as an alternative β-secretase by generating truncated Aβ peptides [6], suggest meprin β as a potential target in AD. As shown *in cellulo* for these substrates propeptide inhibitors could presents promising options for therapeutic intervention. To investigate the inhibitory effect of the propeptide in a more physiological context, we utilized ex vivo cultured brain slices overexpressing meprin β in astrocytes. By applying the propeptide to these cultures, we demonstrated inhibition of endogenously expressed latrophilin-3 and its cleavage by meprin β in this model. This supports the feasibility of using the propeptide in potential in vivo applications, a strategy which has already been successfully implemented for ADAM17. In a tumor mouse model, where ADAM17 is a key driver of metastasis, the prodomain of ADAM17 was intravenously injected and led to decreased metastasis [16]. As shown for the cancer-associated variant meprin β R238Q, the synthetic cognate propeptide inhibitor can easily be optimized to inhibit cancer associated protease variants by the simple exchange of certain amino acids, which may be of interest regarding a personalized anti-cancer-therapy. This might also be a suitable strategy to decrease the interaction of the meprin β propeptide with ovastacin. Overall, peptide-based inhibitors show potential for high target specificity and low off-target activity and can be easily synthesized and modified. However, some limitations of peptide drugs require further discussion, e.g. poor membrane permeability and limited in vivo stability. Despite these disadvantages, peptides have both high affinity and specificity for their biological targets, thereby reducing adverse effects and toxicity. Furthermore, peptides generally exhibit lower immunogenicity, are able to modulate protein–protein interactions and can be produced cost-effectively. Peptide-based inhibitors comprise several options for peptide modifications, that can enhance inhibitory properties and enhance stability [52, 53]. Regarding the use of the meprin β propeptide inhibitor for in vivo application it might be necessary to increase the stability and bioavailability. As demonstrated in this study, the propeptide exhibits limited proteolytic stability toward serine proteases such as trypsin, chymotrypsin, and thrombin; however, its stability may be enhanced by rationally designed amino acid substitutions. Notably, in our in vitro testing the propeptide inhibitor is stable against preincubation with mouse serum, which is important for the options of intravenous injections. This observed stability may result from limited proteolytic activity in the circulation due to endogenous protease inhibitors and supports a potential suitability of our inhibitor for a targeted in vivo applications, which however requires more detailed investigations.

In summary, we characterized and optimized the synthetic propeptide of meprin β as a competitive inhibitor with promising molecular properties in terms of size, specificity, and bioavailability. The translation to in vivo applications in preclinical mouse experiments and clinical trials will be part of future studies.

## Supplementary Information

Below is the link to the electronic supplementary material.

Supplementary Material 1 (DOCX 1.42 MB)

## Data Availability

Not applicable.

## Abbreviations

5-fam: 5-carboxyfluorescein
AD: Alzheimer’s disease
ADAM: a disintegrin and metalloproteinase
ADHD: attention deficit hyperactivity disorder
ANOVA with Bonferroni adjustment: analysis of variance
APP: amyloid precursor protein
BMP1: bone morphogenetic protein 1
dabcyl: Acetyl-dArg(3)-dGlu(3)-hexaminoyl-K-4-((4-(Dimethylamino)phenyl)azo)benzoic
DMEM: Dulbecco's Modified Eagle Medium
DTT: dithiothreitol
FBS: fetal bovine serum
GAPDH: glyceraldehyde 3-phosphate dehydrogenase
GFAP: glial fibrally acidic protein
GPCR: G protein-coupled receptor
HEK293T_A10/17^−/−^: HEK293T cells deficient for ADAM10 and ADAM17
IL-6R: interleukin-6 receptor
KD: dissociation constant
latrophilin-3: latrophilin-3
MAM: meprin/A5 protein/receptor protein tyrosine phosphatase µ
mca: 7-methyloxycoumarin-4-yl
dnp: K-ε-dinitrophenyl
MST: microscale thermophoresis
OBSC: organotypic brain slice culture
PBS: phosphate-buffered saline
PEI: polyethyleneimine
propeptide∆C: C-terminally truncated propeptide
RFU: relative fluorescent unit
SDS: sodium dodecyl sulfate
SDS-PAGE: SDS-polyacrylamide gel electrophoresis
TBS: tris-buffered saline
TRAF: tumor necrosis factor receptor-associated factor

## References

[1] Broder C, Becker-Pauly C (2013) The metalloproteases meprin α and meprin β: unique enzymes in inflammation, neurodegeneration, cancer and fibrosis. Biochem J 450:253–264 10.1042/BJ20121751 PMC3573791 23410038

[2] Wichert R et al (2017) Mucus Detachment by Host Metalloprotease Meprin β Requires Shedding of Its Inactive Pro-form, which Is Abrogated by the Pathogenic Protease RgpB. Cell Rep 21:2090–2103 10.1016/j.celrep.2017.10.087 29166602

[3] Bülck C et al (2023) Proteolytic processing of galectin-3 by meprin metalloproteases is crucial for host-microbiome homeostasis. Sci Adv 9:eadf4055 10.1126/sciadv.adf4055 PMC10065446 37000885

[4] Werny L, Colmorgen C, Becker-Pauly C (2022) Regulation of meprin metalloproteases in mucosal homeostasis. Biochim et Biophys Acta (BBA) - Mol Cell Res 1869:119158 10.1016/j.bbamcr.2021.119158 34626680

[5] Broder C et al (2013) Metalloproteases meprin α and meprin β are C- and N-procollagen proteinases important for collagen assembly and tensile strength. Proc Natl Acad Sci U S A 110:14219–14224 10.1073/pnas.1305464110 PMC3761563 23940311

[6] Bien J et al (2012) The metalloprotease meprin β generates amino terminal-truncated amyloid β peptide species. J Biol Chem 287:33304–33313 10.1074/jbc.M112.395608 PMC3460434 22879596

[7] Marengo L et al (2022) Meprin β knockout reduces brain Aβ levels and rescues learning and memory impairments in the APP/lon mouse model for Alzheimer’s disease. Cell Mol Life Sci 79:168 10.1007/s00018-022-04205-5 PMC8891209 35235058

[8] Arnold P et al (2017) Meprin Metalloproteases Generate Biologically Active Soluble Interleukin-6 Receptor to Induce Trans-Signaling. Sci Rep 7:44053 10.1038/srep44053 PMC5343444 28276471

[9] Kruse M-N et al (2004) Human meprin alpha and beta homo-oligomers: cleavage of basement membrane proteins and sensitivity to metalloprotease inhibitors. Biochem J 378:383–389 10.1042/BJ20031163 PMC1223953 14594449

[10] Kousalová J et al (2022) Metastatic spread inhibition of cancer cells through stimuli-sensitive HPMA copolymer-bound actinonin nanomedicines. Nanomed Nanotechnol Biol Med 44:102578 10.1016/j.nano.2022.102578 35779856

[11] Botos I, Scapozza L, Zhang D, Liotta LA, Meyer EF (1996) Batimastat, a potent matrix mealloproteinase inhibitor, exhibits an unexpected mode of binding. Proc Natl Acad Sci U S A 93:2749–2754 10.1073/pnas.93.7.2749 PMC39703 8610113

[12] Karmilin K et al (2019) Mammalian plasma fetuin-B is a selective inhibitor of ovastacin and meprin metalloproteinases. Sci Rep 9:546 10.1038/s41598-018-37024-5 PMC6346019 30679641

[13] Bickenbach K et al (2025) Targeted approach to determine the impact of cancer-associated protease variants. Sci Adv 11:eadp5958 10.1126/sciadv.adp5958 PMC11818018 39937919

[14] Moss ML et al (2007) The ADAM10 Prodomain Is a Specific Inhibitor of ADAM10 Proteolytic Activity and Inhibits Cellular Shedding Events. J Biol Chem 282:35712–35721 10.1074/jbc.M703231200 17895248

[15] Wong E et al (2016) Harnessing the natural inhibitory domain to control TNFα Converting Enzyme (TACE) activity in vivo. Sci Rep 6:35598 10.1038/srep35598 PMC5159831 27982031

[16] Bolik J et al (2022) Inhibition of ADAM17 impairs endothelial cell necroptosis and blocks metastasis. J Exp Med 219:e20201039 10.1084/jem.20201039 PMC8689681 34919140

[17] Becker-Pauly C et al (2007) The α and β Subunits of the Metalloprotease Meprin Are Expressed in Separate Layers of Human Epidermis, Revealing Different Functions in Keratinocyte Proliferation and Differentiation. J Invest Dermatology 127:1115–1125 10.1038/sj.jid.5700675 17195012

[18] Blanc G et al (2007) Insights into How CUB Domains Can Exert Specific Functions while Sharing a Common Fold. J Biol Chem 282:16924–16933 10.1074/jbc.M701610200 17446170

[19] Eckhard U, Körschgen H, Von Wiegen N, Stöcker W, Gomis-Rüth FX (2021) The crystal structure of a 250-kDa heterotetrameric particle explains inhibition of sheddase meprin β by endogenous fetuin-B. Proc Natl Acad Sci USA 118:e2023839118 10.1073/pnas.2023839118 PMC8040653 33782129

[20] Jerabek-Willemsen M et al (2014) MicroScale Thermophoresis: Interaction analysis and beyond. J Mol Struct 1077:101–113

[21] Riethmueller S et al (2016) Cleavage Site Localization Differentially Controls Interleukin-6 Receptor Proteolysis by ADAM10 and ADAM17. Sci Rep 6:25550 10.1038/srep25550 PMC4858764 27151651

[22] Armbrust F et al (2024) A novel mouse model for N-terminal truncated Aβ2-x generation through meprin β overexpression in astrocytes. Cell Mol Life Sci 81:139 10.1007/s00018-024-05139-w PMC10937767 38480559

[23] Scharfenberg F et al (2020) Degradome of soluble ADAM10 and ADAM17 metalloproteases. Cell Mol Life Sci 77:331–350 10.1007/s00018-019-03184-4 PMC11105009 31209506

[24] Talantikite M et al (2018) Inhibitors of BMP -1/tolloid‐like proteinases: efficacy, selectivity and cellular toxicity. FEBS Open Bio 8:2011–2021 10.1002/2211-5463.12540 PMC6275283 30524951

[25] Werny L et al (2023) MT1-MMP and ADAM10/17 exhibit a remarkable overlap of shedding properties. FEBS J 290:93–111 10.1111/febs.16586 35944080

[26] Lu J, Sun PD (2020) High affinity binding of SARS-CoV-2 spike protein enhances ACE2 carboxypeptidase activity. Preprint at. 10.1101/2020.07.01.182659 PMC7833600 33122196

[27] Sriramula S, Pedersen KB, Xia H, Lazartigues E (2017) Determining the Enzymatic Activity of Angiotensin-Converting Enzyme 2 (ACE2) in Brain Tissue and Cerebrospinal Fluid Using a Quenched Fluorescent Substrate. Methods Mol Biol 1527:117–126 10.1007/978-1-4939-6625-7_9 PMC12884534 28116711

[28] Jin G et al (2002) A continuous fluorimetric assay for tumor necrosis factor-alpha converting enzyme. Anal Biochem 302:269–275 10.1006/abio.2001.5549 11878807

[29] Körschgen H et al (2020) A Primary Evaluation of Potential Small-Molecule Inhibitors of the Astacin Metalloproteinase Ovastacin, a Novel Drug Target in Female Infertility Treatment. ChemMedChem 15:1499–1504 10.1002/cmdc.202000397 PMC7496240 32946206

[30] Kuske M et al (2021) Limited proteolysis by acrosin affects sperm-binding and mechanical resilience of the mouse zona pellucida. Mol Hum Reprod 27:gaab022 10.1093/molehr/gaab022 33779727

[31] Bertenshaw GP et al (2001) Marked Differences between Metalloproteases Meprin A and B in Substrate and Peptide Bond Specificity. J Biol Chem 276:13248–13255 10.1074/jbc.M011414200 11278902

[32] Arolas JL et al (2012) Structural basis for the sheddase function of human meprin β metalloproteinase at the plasma membrane. Proc Natl Acad Sci USA 109:16131–16136 10.1073/pnas.1211076109 PMC3479590 22988105

[33] Kim A-R et al (2024) Enhanced protein-protein interaction discovery via alphafold-multimer 2024.02.19.58097. Preprint at 10.1101/2024.02.19.580970

[34] Copeland RA (2005) Evaluation of enzyme inhibitors in drug discovery. A guide for medicinal chemists and pharmacologists. Methods Biochem Anal 46:1–265 16350889

[35] Armbrust F et al (2025) HYTANE-Identified Latrophilin-3 Cleavage by Meprin β Leads to Loss of the Interaction Domains. J Proteome Res 24:1832–1844 10.1021/acs.jproteome.4c00912 PMC11976865 40135725

[36] Wiltfang J et al (2001) Elevation of β-Amyloid Peptide 2–42 in Sporadic and Familial Alzheimer’s Disease and Its Generation in PS1 Knockout Cells. J Biol Chem 276:42645–42657 10.1074/jbc.M102790200 11526104

[37] Asai M et al (2003) Putative function of ADAM9, ADAM10, and ADAM17 as APP alpha-secretase. Biochem Biophys Res Commun 301:231–235 10.1016/s0006-291x(02)02999-6 12535668

[38] Biasin V et al (2017) Meprin β contributes to collagen deposition in lung fibrosis. Sci Rep 7:39969 10.1038/srep39969 PMC5216360 28059112

[39] Banerjee S, Bond JS (2008) Prointerleukin-18 Is Activated by Meprin β in Vitro and in Vivo in Intestinal Inflammation. J Biol Chem 283:31371–31377 10.1074/jbc.M802814200 PMC2581578 18786924

[40] Gomis-Rüth FX, Stöcker W (2022) Structural and evolutionary insights into astacin metallopeptidases. Front Mol Biosci 9:1080836 10.3389/fmolb.2022.1080836 PMC9848320 36685277

[41] Mathew R et al (2005) Meprin-α in chronic diabetic nephropathy: interaction with the renin-angiotensin axis. Am J Physiology-Renal Physiol 289:F911–F921 10.1152/ajprenal.00037.2005 15942051

[42] Felten M et al (2024) Substrate profiling of the metalloproteinase ovastacin uncovers specific enzyme–substrate interactions and discloses fertilization-relevant substrates. FEBS J 291:114–131 10.1111/febs.16954 37690456

[43] Burkart AD, Xiong B, Baibakov B, Jiménez-Movilla M, Dean J (2012) Ovastacin, a cortical granule protease, cleaves ZP2 in the zona pellucida to prevent polyspermy. J Cell Biol 197:37–44 10.1083/jcb.201112094 PMC3317803 22472438

[44] Chen DZ et al (2000) Actinonin, a Naturally Occurring Antibacterial Agent, Is a Potent Deformylase Inhibitor. Biochemistry 39:1256–1262 10.1021/bi992245y 10684604

[45] Wojtowicz-Praga SM, Dickson RB, Hawkins MJ (1997) Matrix metalloproteinase inhibitors. Invest New Drugs 15:61–75 10.1023/a:1005722729132 9195290

[46] Tan K et al (2023) Synthesis and structure–activity relationships of pyrazole-based inhibitors of meprin α and β. J Enzyme Inhib Med Chem 38:2165648 10.1080/14756366.2023.2165648 PMC9870012 36661029

[47] Linnert M et al (2021) Structure and Dynamics of Meprin β in Complex with a Hydroxamate-Based Inhibitor. Int J Mol Sci 22:5651 10.3390/ijms22115651 PMC8197800 34073350

[48] Ramsbeck D et al (2018) Structure-Guided Design, Synthesis, and Characterization of Next-Generation Meprin β Inhibitors. J Med Chem 61:4578–4592 10.1021/acs.jmedchem.8b00330 29694039

[49] Ramsbeck D, Hamann A, Schlenzig D, Schilling S, Buchholz M (2017) First insight into structure-activity relationships of selective meprin β inhibitors. Bioorg Med Chem Lett 27:2428–2431 10.1016/j.bmcl.2017.04.012 28408220

[50] Fields GB (2019) The Rebirth of Matrix Metalloproteinase Inhibitors: Moving Beyond the Dogma. Cells 8:984 10.3390/cells8090984 PMC6769477 31461880

[51] Rose-John S (2012) IL-6 Trans-Signaling via the Soluble IL-6 Receptor: Importance for the Pro-Inflammatory Activities of IL-6. Int J Biol Sci 8:1237–1247 10.7150/ijbs.4989 PMC3491447 23136552

[52] Lau JL, Dunn MK (2018) Therapeutic peptides: Historical perspectives, current development trends, and future directions. Bioorg Med Chem 26:2700–2707 10.1016/j.bmc.2017.06.052 28720325

[53] Wang L et al (2022) Therapeutic peptides: current applications and future directions. Sig Transduct Target Ther 7:48 10.1038/s41392-022-00904-4 PMC8844085 35165272

