# Supplementary material for: Meprin β is specifically inhibited by its cognate synthetic propeptide
Source: Cell Mol Life Sci. 2026 Jul 23;83(1):385. doi: 10.1007/s00018-026-06361-4 (PMC13680589; doi:10.1007/s00018-026-06361-4)
Supplement: Supplementary file 1 [file 18_2026_6361_MOESM1_ESM.docx]

**Supplementary Material:**

**Meprin β is specifically inhibited by its cognate synthetic propeptide**

## Fred Armbrust^1*^, Hannah Bruhn^1*^, Kira Bickenbach^1*^, Nicolas Steen^1^, Sapir Shcoory^2^, Nikolaos Afratis^2^, Nele Korn^1^, Hagen Körschgen^3^, Catherine Moali^4^, Claus Pietrzik^5^, Irit Sagi^2+^ and Christoph Becker-Pauly^1+^

^1^ Biochemical Institute, Unit for Degradomics of the Protease Web, Kiel University, Kiel, Germany,

^2^ Weizmann Institute of Science, Dept. of Immunology and Regenerative Biology, Rehovot, Israel

^3^ Institute for Pathobiochemistry, University Medical Center of the Johannes Gutenberg University of Mainz, Mainz, Germany

^4^ Tissue Biology and Therapeutic Engineering Laboratory (LBTI-UMR 5305 CNRS), Lyon University, Lyon, France

^5^ Molecular Neurodegeneration, Institute for Pathobiochemistry, University Medical Center of the Johannes Gutenberg University of Mainz, Mainz, Germany

^*^ Contributed equally

^+^Correspondence:

Prof. Dr. Irit Sagi, Weizmann Institute of Science, Dept. of Immunology and Regenerative Biology, Max and Lillian Candiotty Building, Room 207, 234 Herzl St. Rehovot, 76100, Israel, Tel: 00972-8-934 2130, E-mail: irit.sagi@weizmann.ac.il

and

Prof. Dr. Christoph Becker-Pauly, Biochemical Institute, Unit for Degradomics of the Protease Web, University of Kiel, Otto-Hahn-Platz 9, 24118 Kiel, Germany, Tel: 0049-431-880 7118, Fax: 0049-431-880 2238, E-mail: cbeckerpauly@biochem.uni-kiel.de

**Supplementary Fig. S1: Raw data of the MST measurement to evaluate the interaction of meprin β and exogenously added propeptide. (A) (B)** MST signal of fluorescent labelled active meprin β (A) or promeprin β (B) upon binding of the propeptide as ligand. (B) Change in MST signal defined as the change in the normalized fluorescence (Fnorm), which is defined as F_1_/F_0_ and shown for different concentrations of the propeptide ligand (6 pM to 50 µM). (F_1_: fluorescence after a given MST-on time, F_0_: fluorescence prior to IR laser activation). Change of MST in dependence of ligand concentrations is shown in a dose-response curve, which was fitted to derive binding constants.

**Supplementary Fig. S2: Meprin β inhibition by the propeptide on HEK ADAM10/17^-/-^ cells. (A), (B)** HEK ADAM 10/17^-/-^ cells transfected with meprin β were preincubated with different concentrations of its synthetic propeptide, actinonin or fetuin-B. Meprin β activity at the cell surface was measured using a quenched fluorescent peptide substrate (n = 3).

**Supplementary Fig. S3: Analysis of inhibitory capacity of the synthetic C-terminal truncated propeptide of meprin β towards other metalloproteases. (A-G)** Recombinant active metalloproteases (ACE1 (5 nM) (**A**), ACE2 (5 nM) (**B**), ADAM17 (5 nM) (**C**), MMP1 (5 nM) (**D**), MMP2 (5 nM) (**E**), MMP14 (5 nM) (**F**), ovastacin (20 nM)) (**G**) were preincubated with different concentrations of the synthetic C-terminally truncated propeptide of meprin β (propeptide∆C). Protease activities were measured using specific quenched fluorogenic peptide substrates (n = 3). Significances were determined by one-way ANOVA with Bonferroni adjustment (ns: p ≥ 0.05; *: p < 0.05, **: p < 0.01, ***: p < 0.001).

**Supplementary Fig. S4: Stability of the synthetic C-terminal truncated propeptide of meprin β towards pH, temperature, and proteolytic processing . (A)** Propeptide∆C was preincubated at indicated temperatures for 20 min prior to incubation with recombinant meprin β. Meprin β activity (5 nM meprin β) was measured using a quenched fluorogenic peptide substrate (n = 3). The significance was determined by two-way ANOVA with Bonferroni adjustment (***: p < 0.001). **(B)** Propeptide∆C was preincubated in PBS with indicated pH for 20 min prior to dilution to reach pH 7.4 and incubation with recombinant meprin β. As uninhibited control pH-buffers without addition of propeptide∆C were used. Meprin β activity (5 nM meprin β) was measured using a quenched fluorogenic peptide substrate (n = 3). The significance was determined by two-way ANOVA with Bonferroni adjustment (***: p < 0.001). **(C)** Propeptide∆C was freeze-thawn for indicated number of cycles prior to incubation with recombinant meprin β. Meprin β activity (5 nM meprin β) was measured using a quenched fluorogenic peptide substrate (n = 3). The significance was determined by two-way ANOVA with Bonferroni adjustment (***: p < 0.001). **(D)** Propeptide∆C was preincubated with trypsin, chymotrypsin or thrombin using indicated concentrations for 20 min. Afterwards these proteases were inhibited and the reaction mixture was incubated with recombinant meprin β. Furthermore, propeptide ∆C was preincubated with mouse serum. As a control incubation conditions without propeptide∆C (uninhibited) were used. Meprin β activity (5 nM meprin β) as measured using a quenched fluorogenic peptide substrate (n = 3). The significance was determined by two-way ANOVA with Bonferroni adjustment (*: p < 0.05, ***: p < 0.001).
